# Drug-Drug Interaction of Ergotamine with a Combination of Darunavir, Abacavir, and Lamivudine Causing a Fatal Vasospastic Ischemia

**DOI:** 10.1155/2018/4107450

**Published:** 2018-12-19

**Authors:** Elisabeth Petit, Karen Schoonheydt, Philippe Meert, Marie Van Laer

**Affiliations:** ^1^Université Catholique de Louvain, Place de l'Université 1, 1348 Louvain-la-Neuve, Belgium; ^2^ZNA Stuivenberg, Lange Beeldekensstraat 267, 2060 Antwerpen, Belgium

## Abstract

Ergotamine toxicity has become a rare condition which can be caused by, among others, drug-drug interaction. In this work we report a case with vasospastic ischemia induced by the wrongful combination of ergotamine with recently started Antiretroviral Therapy. Clinicians were not aware that patient was self-medicating for years with medication containing ergotamine and caffeine for migraines. This diagnosis was established after evaluating the evolving ‘and spreading' ischemia and CT scans and thoroughly interviewing patient's family. Treatment was started with intravenous nimodipine and intra-arterial sodium nitroprusside on the affected limbs. The patient developed severe limb ischemia, cerebral ischemia, and metabolic encephalopathy. Unfortunately no improvements were noticeable and due to evolving cerebral edema as a result of the ischemia, the patient developed brain herniation and died shortly after.

## 1. Introduction

Ergotism or ergotamine toxicity, denoted as ET, is an important and nowadays rare condition [1, 2]. Ergotamine mimics the neurotransmitters norepinephrine, serotonin, and dopamine and can result in smaller and bigger vessels vasospasm and vasoconstriction [3]. Symptoms of ET can be nausea, vomiting, abdominal pain, agitation, hallucinations, lethargy, confusion, painful spasms, seizures, feeble peripheral pulses and cyanotic limbs, loss of peripheral sensation, edema, and gangrene of affected tissues [4]. In some cases vasospasm can also occur in the blood vessels of the brain, kidney, eye, heart, and intestines [2]. If toxicity is left untreated or diagnosed late, it can eventually cause necrotising limbs leading to amputations [5], irreversible coma [6], or even possible dead [7].

The cause of ET is often ascribed to the ingestion of specific alkaloids (e.g., ergotamine and dihydroergotamine) produced by the* Claviceps purpurea* fungus as result of consumption of grain infested by fungi [2, 3, 8]. Furthermore it can also be induced after the administration of medication containing ergotamine [4, 7, 9]. Even in therapeutic doses it can cause ET in susceptible individuals, for example, those with peripheral vascular disease, coronary artery disease, infection, or renal/hepatic impairment [1, 2, 5]. Moreover ET can be caused by accidental overdosing (> 0.25mg/kg) or by drug-drug interactions [4] including macrolide antibiotics [10], azole antifungals, HIV protease inhibitors, and some antidepressants [1, 6, 11].

When faced with symptoms compatible with vasospasm or nonocclusive arterial ischemia and in absence of any thrombophilic pathology or arteritides, thorough interview and physical examination are important to find clues to this rare disorder. In addition, it is crucial to differentiate with more common causes of limb ischemia with the use of CT angiography.

To emphasis the importance of ET as a result of drug-drug interaction, this report describes the case of a middle-aged woman who died of ET by recently started Antiretroviral Therapy (ART) and Cafergot® (medication containing of ergotamine and caffeine used in the treatment of migraine). This relatively rare disease is difficult to recognize and treatment is sometimes ambiguous.

## 2. Case Report

### 2.1. Description Patient

We report a case of a 50-year-old woman, normal weight and length (49 kg, 164 cm), known to have HIV for the previous 6 years and who was recently started on ART (2 weeks ago). No report was found of the patient taking any HIV therapy in the past. The ART started was abacavir-lamivudine, respectively, 600mg and 300 mg once daily in combination with darunavir 400mg twice daily. Further medical history consists of depression and anxiety, migraines, gastroesophageal reflux disease, and no known use of illicit drugs. She was admitted in the Intensive Care Unit (ICU) after being apathetic, lethargic, and having a painful cold cyanotic right hand for 4 days. The day of hospitalization she was found very drowsy on the floor by her partner who called the health emergency services immediately. Earlier that week a general practitioner was consulted concerning the painful hand. He suggested this was due to lateral epicondylitis and treated her with NSAID's.

### 2.2. Clinical Examination

In hospital on admission physical examination revealed a woman with a Glasgow Coma Scale of 13/15, conscious but sleepy, oriented, slow speech, and grossly intact cranial nerves. On examination she had cyanotic cold toes on the right foot and a cyanotic right hand extending to the wrist and no pulsations were felt on all 4 limbs (no sign of edema). The patient had a blood pressure of 140/70 mmHg on the left arm and heart rate of 80 beats/min. On auscultation no irregularities were found and the SpO^2^ indicated 99%. Examination revealed no abdominal abnormalities, but auscultation showed hyperactive bowel sounds. There were no signs of a fever and the urine output was normal. On admission the SAPS score was 40, APACHE-II score 12, MODScore 6, and SOFA score 4; thus the mortality prediction by the SAPS-II score was 24.7%.

### 2.3. Investigations

A central venous catheter was inserted at the Emergency Department due to failed attempts of placing a peripheral canula. Complete blood cell count showed a white blood count of 11.5 x 10^9^/L and no further abnormalities (see supplementary information nr 1). The serum biochemistry showed a CRP of 24 mg/dl, CK 2285U/L, normal renal function with a of sodium of 121 mEq/L and chloride 87 mEq/L. Lactate was 0.7 mmol/L and troponins were negative with two consecutive blood samples. Toxicology screening, including ethanol, were also negative. Coagulation showed normal PT and APTT but D-dimers were raised to 2.2 mg/L (reference normally < 0.5). HIV viral load showed 42 copies/mL and CD4 count was 380 cells per cubic millimeter of blood.

Because of the altered consciousness and lethargy a CT scan of the head was done. This showed no acute intracranial abnormality. During the following days the patient deteriorated and showed increasing unconsciousness and ischemic limbs. Platelet and coagulation disorders were excluded and the lumbar puncture showed no abnormalities. On the 3^th^ day of admission, due to quickly lowering Glasgow coma scale, the patient needed intubation. At the same day a CT angiography of the aorta and lower limbs was done to find a cause for the increasingly cyanotic limbs. Findings included (i) multiple renal infarctions, (ii) narrowing of the external iliac arteries, and (iii) bilateral narrowed femoral-popliteal arteries with multitudinous stenosis or occlusions. The radiologists had difficulties to differentiate between thrombogenic pathology, medically induced arterial spasms, hypovolemia, or congenital hypotrophic arteries.

**Figure 1:**
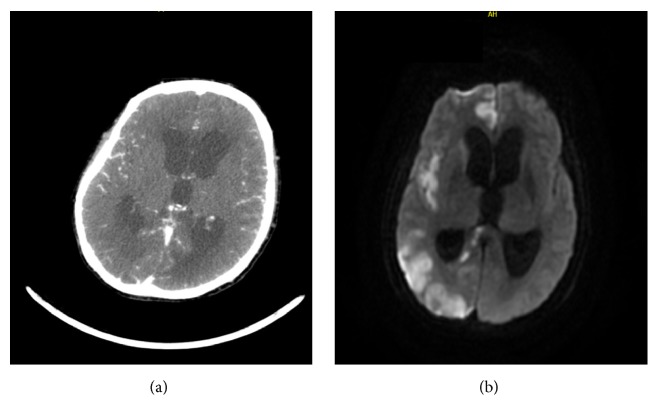
(a) CT angiography of the head showing important supratentorial hydrocephalus with narrowing of cortical sulci and cerebral edema. (b) MRI-scan of the head showing extensive recent ischemia in the cerebellum and dilatation of the third and fourth ventricle.

Two days after intubation, when sedation was stopped, clinical examination showed no improvement of consciousness and fixed pupils were noticed. A CT angiography of the head (Figure 1(a)) showed important supratentorial hydrocephalus with narrowing of cortical sulci and cerebral edema. A normal intracranial perfusion, caliber, and patency of the vertebral arteries and the carotid arteries were seen.

On the 6^th^ day, a head MRI (Figure 1(b)) showed extensive recent ischemia in the cerebellum and hemispheres, more pronounced on the right side than the left side. An obstructive dilatation of the third and fourth ventricle with clear signs of edema in the posterior fossa and pontocerebellar cisterna could be noticed. In addition, beginning herniation of the cerebral tonsils in the foramen magnum was visible. The MRI showed flow void in the basilar arteria and internal carotid arteria. Blood results had shown a slow decline in red blood cell count to 2.74 x 10^12^/L with a hemoglobin of 8.2 g/dl and a rise in white blood cell count up to 17.8 x 10^9^/L with CRP of 27 mg/dl on the day she passed away. Liver function tests and renal function were slightly elevated.

### 2.4. Treatment

After seeing the evolving ischemia with several CT's that could not clearly differentiate between nonocclusive vasospasms or multitudinous stenosis and no sign of underlying peripheral artery disease, the medical history of the patient was re-examined. This revealed a document 10 years prior to the current hospitalisation suggesting former use of ergotamine containing medication (Cafergot®). Current use of ergotamine prior to hospitalization for a severe migraine attack was also confirmed by the partner. All this information in combination with the clinical presentation led to the diagnosis of ET.

Treatment was promptly started (3 days after admission), with IV nimodipine, intra-arterial sodium nitroprusside, and nitroglycerin transdermal patches on affected limbs (both legs and right arm). In addition, an epidural catheter was placed for infusion of bupivacaine. The HIV medication was discontinued and to prevent secondary thrombosis anticoagulation with low-molecular-weight heparin was started. However, no improvement was noticeable.

### 2.5. Outcome

Two days after stopping sedation, the patient did not wake up and pupils were fixed. MRI of the head (Figure 1(b)) showed beginning herniation of the cerebral tonsils in the foramen magnum. Neurosurgeons believed that the brain damage due to hydrocephalus was irreversible and that there would be no improvement with external ventricular drainage. Due to the evolving symptomatology and poor prognosis, in agreement with family futile life sustaining therapies were limited and supportive therapy for comfort was started. Patient died 7 days after being admitted at the ICU.

## 3. Discussion

Toxicity with ergotamine can be caused by (i) grain infested fungi, (ii) ergotamine containing medication or (iii) drug-drug interaction. Drugs that inhibit CYP3A4 [1, 6, 7, 11, 12], among them darunavir and other protease inhibitors, can raise the ergotamine concentration to toxic levels, even when ergotamine is administered at low doses [2, 6] (in this case patient was not taking any other medication which could interact with CYP3A4). The clinical presentation of ET depends upon underlying comorbidities and the time course of ergotamine administration. The latter is difficult to identify due to wide variety in symptomatology and broad differential diagnosis for ischemic limbs. In this case the patient was self-medicating with ergotamine and caffeine in normal therapeutic doses for migraine in combination with recently started darunavir as part of ART when she started to show symptoms of ergotism.

First-line treatment of ET is to discontinue the causative agent or medication (half-life of 2-2.5 hours) [13]. In most cases, findings will generally be seen to improve [1, 5, 11, 12]. Nevertheless, pharmacological vasodilation therapy is often necessary to improve the symptomatology of the patient. However, to the best of our knowledge, there is no consensus in how to treat patients with ET as it is a rare disease and no extensive studies have been carried out [1, 2]. Treatment methods are mainly based on other vasospastic illnesses and case reports [7, 11, 12]. Some literature describes invasive treatments such as epidural spinal cord stimulation and balloon angioplasty but caution must be exercised as they risk damaging otherwise normal arteries [1, 2, 5, 10]. Prophylaxis to prevent vasospasm-induced stasis and thrombosis is widely accepted and used in ET. In this case, medical treatment was ineffective due to late presentation of the patient in the hospital, the severity of presented ischemia, and toxicity having been diagnosed only 3 days after patient was hospitalized and continued to deteriorate.

Rey et al. [6] reported a similar case with use of ritonavir where medical treatment was ineffective. It was reported that the patient stayed in an irreversible coma. Another case report [7] shows the death of a young female after taking ergotamine in combination with indinavir (in this case not many details about the case are known and clinical presentation was described as hypoxia and shock). To our knowledge these two cases are the only ones which prescribe a deadly outcome after drug-drug interaction of protease inhibitors and ergotamine though many case reports are described with better outcomes. Avihingsanon et al. reported [7] 23 cases of clinical ergotism associated with use of HIV-protease inhibitors. In 78.3 % of patients full recovery was seen, but in the remaining 21.7 % of patients amputation, gangrene, and 1 death occurred. Orrapin et al. [12] reported 4 cases; 2 of them showed full recovery, 1 patient had a Chopart amputation and the last had dry gangrene of distal phalanx, resulting in the autoamputation of both upper and lower extremities. Fröhlich et al. [11] identified 11 similar cases after searching Pubmed between 1997 and 2010; 8 patients showed full recovery, with one patient being the case of Rey et al. [6], a second patient suffering from right-sided central peroneal nerve paresis, and the 3^th^ hjaving a transmetatarsal amputation.

Deadly outcome or severe sequelae resulting from drug-drug interaction of protease inhibitors and ergotamine could be avoided if patients and healthcare professionals were to be better informed about possible drug-drug interactions especially when using ART. Avihingsanon et al. [7] suggested that patients do not always inform their pharmacist and health care workers about using HIV treatment due to potential stigma. Automated surveillance systems or online platforms for prescribed medication or purchased over-the-counter medication, could help pharmacists alerting patients. Several authors highlight the importance of thoroughly interviewing patients about their medication, self-administered medication and use of over-the-counter medication, especially when being confronted with medication that is known for having severe drug-drug interactions [5, 9–12].

Unfortunately, in some countries medication containing ergotamine is still available over-the-counter. The worldwide use of ergotamine is in decline as sumatriptan and other triptans became the golden standard to stop a migraine attack as NSAID's also proved to be effective [9, 14].

## 4. Conclusion

This work reports a case of an adult woman, hospitalized for lethargy and an ischemic limb. Initially no diagnosis was made. Nevertheless after looking further into her medical history use of ergotamine containing medication (Cafergot®) for migraines was noticed. This, in combination with ART which only started 2 weeks prior to this hospitalization, is known to cause severe forms of ergotism. Unfortunately, no improvement was observed after treatment and, five days after being admitted, the patient developed cerebral edema and brain herniation. Due to poor prognosis, treatment was changed to comfort therapy and the patient died a few days later. To avoid similar cases in the future, medical personal should always take into account that patients could use over-the-counter medication or could have been using medication over a period of years without informing their healthcare professional. When being started on antiretroviral medication patients should be informed about potentially harmful drug-drug interactions.

## Supplementary Materials

Supplementary Materials The supplementary information consists out of 2 tables. (1) Supplementary information nr. 1 (Table 1) contains the blood results at the time the patient was admitted to the Emergency Department and afterwards at the ICU. In addition, supplementary nr. 1 also gives an overview of the day-to-day results for hematology and biochemistry to show the evolution of different blood results while the patient was at ICU. (2) Supplementary information nr. 2 (Table 2) contains the additional urine results taken at the first day of admission.
