# Supplementary material for: Drug-Drug Interaction of Ergotamine with a Combination of Darunavir, Abacavir, and Lamivudine Causing a Fatal Vasospastic Ischemia
Source: Case Rep Emerg Med. 2018 Dec 19;2018:4107450. doi: 10.1155/2018/4107450 (PMC6313968; doi:10.1155/2018/4107450)
Supplement: Supplementary Materials — The supplementary information consists out of 2 tables. (1) Supplementary information nr. 1 (Table 1) contains the blood results at the time the patient was admitted to the Emergency Department and afterwards at the ICU. In addition, supplementary nr. 1 also gives an overview of the day-to-day results for hematology and biochemistry to show the evolution of different blood results while the patient was at ICU. (2) Supplementary information nr. 2 (Table 2) contains the additional urine results taken at the first day of admission. [file 4107450.f1.pdf]

Supplementary information nr 1: Blood results

	27/08 07:43	27/08 11:51	27/08 17:34	28/08 07:05	29/08 07:03	30/08 07:08	31/08 07:20	01/09 07:21	02/09 07:02	03/09 06:37	Units	Normal values
Hematology												
Haemoglobine	15.1 +		12.3	11.6	9.2 -	9.3 -	10.1 -	9.2 -	9.2 -	8.2 -	g/dl	10.8-14.2
Hematocrit	0.417 +		0.355	0.336 -	0.264 -	0.271 -	0.287 -		0.277 -	0.243 -	l/l	0.35-0.41
Red blood cell count	4.98 +		4.16	3.92	3.07-	3.22 -	3.42 -		3.13 -	2.74 -	10*12/l	3.65-4.59
Reticulocytes	6.20 +			61.50	51.00	62.50	46.80		36.90	59.20	10*9/l	16.0-66.0
Mean cell volume	83.7		85.3	85.7	86.0	84.2	83.9		88.5	88.7	fl	82.4-97.3
Mean cell haemoglobin	30.3		29.6	29.6	30.0	28.9	29.5		29.4	29.9	pg	25.0-34.2
Mean cell haemoglobin concentration	36.2		34.6	34.5	34.8	34.3	35.2		33.2	33.7	g/dl	33.2-36.9
RDW	14.0		14.0	14.0	14.0	14.0	15.0 +		16.0 +	16.0 +	%	12.2-14.8
Platelets	109.0 -		83.0 --	82.0 -	84.0 -	93.0 -	132.0 -	146.0	138.0 -	107.0 -	10*9/l	142.00-330.00
White blood cells	11.5 +		9.3	7.8	6.2	6.6	17.7 +	16.4 +	17.8 +	14.0 +	10*9/l	3.45-9.76
Prothrombin time	82.0			85.0	84.0	84.0	83.0	83.0	66.0 -		%	70.0-100.0
Prothrombin time	14.20			13.90	14.00	14.00	14.10	14.10	16.50		sec	11.0-15.5
APTT	34.8			41.2 +	39.1	50.4 +	37.4	33.1	35.4		sec	28.0-40.0
Fibrinogen	6.60 ++										g/l	2.00-4.00
D-dimer	2.2 ++										mg/l	< 0.5
Biochemistry												
C-reactive Protein	24.1 +		27.8 +	26.9 +	29.0 +	32.8 +	25.0 +	12.1 +	16.9 +	27.2 +	mg/dl	< 1.0
Total protein	67			47 -	38 -	38 -	35 -		45 -	39 -	g/l	60-78
Albumin				2.2 -	1.7 -	2.0	1.5 -		2.4 -	2.0 -	g/l	3.5-5.0
Triglycerides						104			87		mg/dl	< 150
CK	2258 +		4440 +	6398 +	1514 +	1186 +	1532 +		3872 +	2965 +	U/l	35-230
CK-MB	77		113	172	74	41	46		42	24	U/l	
AST	107 +			260 +	136 +	102 +	109 +		194 +	165 +	U/l	14-36
ALT	35			72 +	73 +	67 +	57 +		86 +	87 +	U/l	< 41

LDH	693 +			966 +	1171 +	1050 +	999 +		1428 +	1296 +	U/l	316-618
Amylase	39			55	< 30	< 30	62		115 +	179 +	U/l	30-110
Lipase	36			89	35	35	45		103	128	U/l	23-300
Alk. phosphatase	104			80	77	82	107		121	123	U/l	38-126
Gamma-GT	25			18	29	30	60 +		170 +	187 +	U/l	12-43
Bilirubin				0.1 -	0.2	0.9	0.3		0.5	0.7	mg/dl	0.2-1.3
Glucose	114 +		89	144 +	135 +	98	124 +	132 +	135 +	123 +	mg/dL	76-110
Sodium	121 --	123 --	125 --	124 -	123 -	137	139	138	144	148 +	meq/l	137-145
Potassium	4.0	3.7	4.0	3.9	3.6	4.2	4.4	4.4	4.1	4.1	meq/l	3.5-5.1
Chloride	87 -	89 -	99	95 -	92 -	105	110 +	109 +	111 +	115 +	meq/l	98-107
Bicarbonate	27.9		20.4 -	28.8	31.0 +	28.5	23.5	24.0	25.4	27.8	meq/l	22.0-30.0
Calcium				7.50 -	7.20 -	7.98 -	8.14 -		8.37 -	8.51 -	mg/dl	8.60-10.0
Osmolaliteit				258 -	258 -	283	291		305	320 +	mosm/kg	278-305
Anion-gap	10.1		9.6 -	4.1 -	3.6 -	7.7 -	9.9 -		11.7	9.3 -	meq/l	10.0-18.0
Phosphate			3.53	2.62	1.90 -	1.78 -	2.24 -	1.85 -	2.57	3.17	mg/dl	2.50-4.50
Magnesium			1.43 -	1.73	2.00	2.31 +	2.41 -	2.42 +	2.24	2.52 +	mg/dl	1.60-2.30
Ammonia		22.0									umol/l	11.0-32.0
Urea	24		23	24	24	23	38 +	54 +	64 +	91 +	mg/dl	15-37
Creatinine	0.79		0.77	0.77	0.80	0.86	0.96	1.00	1.02	1.06	mg/dl	0.5-1.2
Estimated GFR	78		80	80	77	70	62	59	58	55	mL/min/1.73m^2	
Uric Acid	3.41			2.69	2.72	2.51	2.89				mg/dl	2.5-7.5
Lactate	0.7										mmol/L	Venous 1.0-2.4
Procalcitonin			0.5								ng/mL	< 0.5
Troponin	< 0.012	<0.012									ng/mL	> 0.12 suggestive for MI
Serology												
Hepatitis B surface antigen		neg										
Hepatitis B core antibodies		neg										

Hepatitis C surface antibodies		neg										
Anti-HAV IgM		doubtfull										
HIV Viral load		42									Copies/ml pl	*
HIV Viral load		1.62									logs	
Syphilis Ig-total			neg									
Anti-cardiolipin IgG			0.2								GPL Units	< 10
Anti-cardiolipin IgM			4.1								MPL units	< 10
Specific Hematology												
Lymphocytes		6.50%										
CD45 events		38119									Pan leucocytes	
Lymphocytes events		2584										
CD3 events		1838									T-lymphocytes	
CD4 events		380									T-helper cells	
CD8 events		1273									T-suppressor	
CD19 events		255									B-lymphocytes	
CD3		4.82									% of T-lymphocytes	
CD4		1.00									% T-helper leucocytes	
CD4		14.71									% T-helper lymphocytes	
CD8		3.34									% T-suppressor leucocytes	
CD19		0.67									% B-lymphocytes	

B-lymphocytes		0.0603									10*9/l CD19+	
Protein C			84.0								%	70-130.0
Protein S			54								%	50-134.0
Beta-2 microglobuline						1.99 +					mg/L	0.70-1.30

*Used test is Amliprep/Cobas Taqman HIV- ROCHE (version 2.0)

Supplementary information nr 2: Additional result (Urine)

Urine results	27/08 12:31	Units	Normal values
pH	6.0		4.8-7.4
Sodium	101.8	meq/L	
Potassium	44.6	meq/l	
Osmolality	579	mosm/kg	300-900
Protein	0.89 ++	g/l	< 0.12
Glucose	neg		+ if > 50mg/dl
Ketones	+	mg/dl	+ if > mg/dl
Urobilinogen	neg		+ if > 1mg/dl
Bilirubin	neg		+ if > 1mg/dl
Nitrite	neg		
Red blood cells	262 +	/microl	0-25
Leucocytes	61 +	/microl	0-25
Epithelial cells	23	/microl	+ if > 30cells/microl
